# Supplementary material for: Rapid single-wavelength lightsheet localization microscopy for clarified tissue
Source: Nat Commun. 2019 Oct 18;10:4762. doi: 10.1038/s41467-019-12715-3 (PMC6800451; doi:10.1038/s41467-019-12715-3)
Supplement: Supplementary file 2 — Supplementary Information [file 41467_2019_12715_MOESM2_ESM.pdf]

Supplementary Information

Rapid single-wavelength lightsheet localization microscopy for clarified tissue

Chu et al.

Supplementary Fig. 1

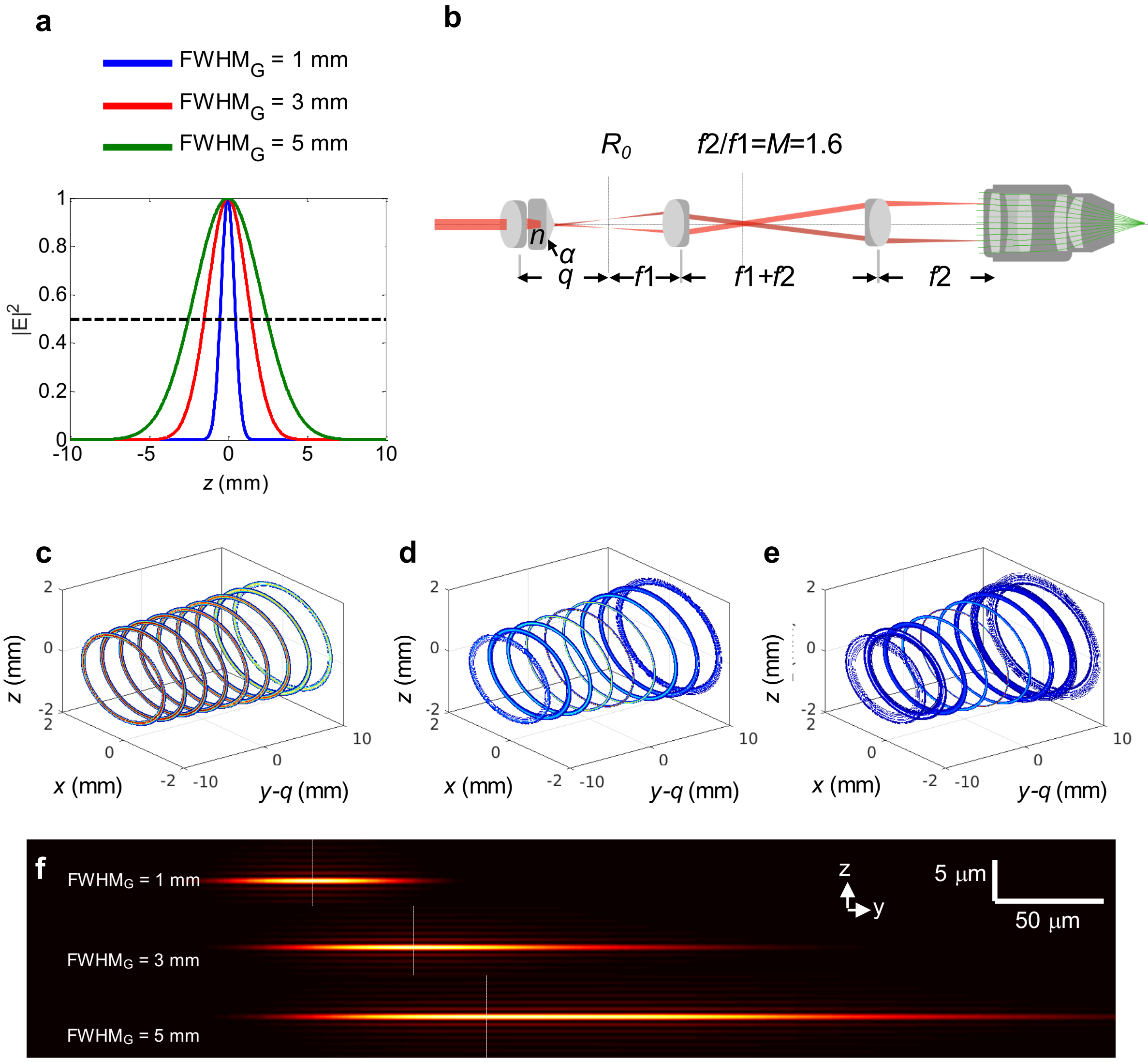

Supplementary Fig. 1. Bessel beam length adjustment using lens-axicon combinations

(a) Intensity profiles of Gaussian beams incident onto the lens-axicon combination. The full-width-half-maxima (FWHM) of Gaussian beams ($FWHM_G$) were 1, 3 and 5 mm, respectively, and the radius of curvature, R_F, for each of their wave-fronts at the lens was set to −813 mm (a converging beam). **(b) A** schematic figure of lens-axicon combination conjugated to the back aperture of an excitation objective lens through a *4f* configuration. The simulation parameters were as follows: laser wavelength λ=637 nm, focal length F=200 mm, axicon angle α=1°, refractive index of the axicon n=1.51, image distance from the ring behind the axicon-lens combination q=160 mm (estimated from the lens law: $F^{-1}= R_F^{-1}+q^{-1}$), ring radius $R_0=\alpha(n-1)q$=1.44 mm, inner diameter of the ring aperture (ID)=2.78 mm, outer diameter of the ring aperture (OD)=2.99 mm, beam expansion ratio after *4f* configuration M=1.6, focal length of the objective lens F_o=7.25 mm and refractive index of the immersion solution n_{im}=1.38. **(c)** Intensity profiles of focused ring patterns at $FWHM_G$ = 1, 3 and 5 mm **(c-e), respectively,** around the image plane (y − q = 0, where q is the image distance). While the three ring patterns have the same diameter on the image plane, the one originating from the Gaussian beam with the largest $FWHM_G$ exhibited the least ring width but the shortest depth of focus around the image plane. **(f)** Intensity profiles of the Bessel beams generated from the ring patterns on the image plane in (c-e). The dashed white lines indicate the positions of highest intensity. While all the Bessel beams have a FWHM beam width of about 0.55 μm, a thin ring width (larger $FWHM_G$) may significantly prolong the beam length along the propagation direction. The FWHMs along the propagation direction are 65, 120 and 220 μm at $FWHM_G$ = 1, 3 and 5 mm, respectively. The Bessel beams also were shifted farther away from the focal plane of the final objective lens as the $FWHM_G$ of the Gaussian beam turns larger.

Supplementary Fig. 2

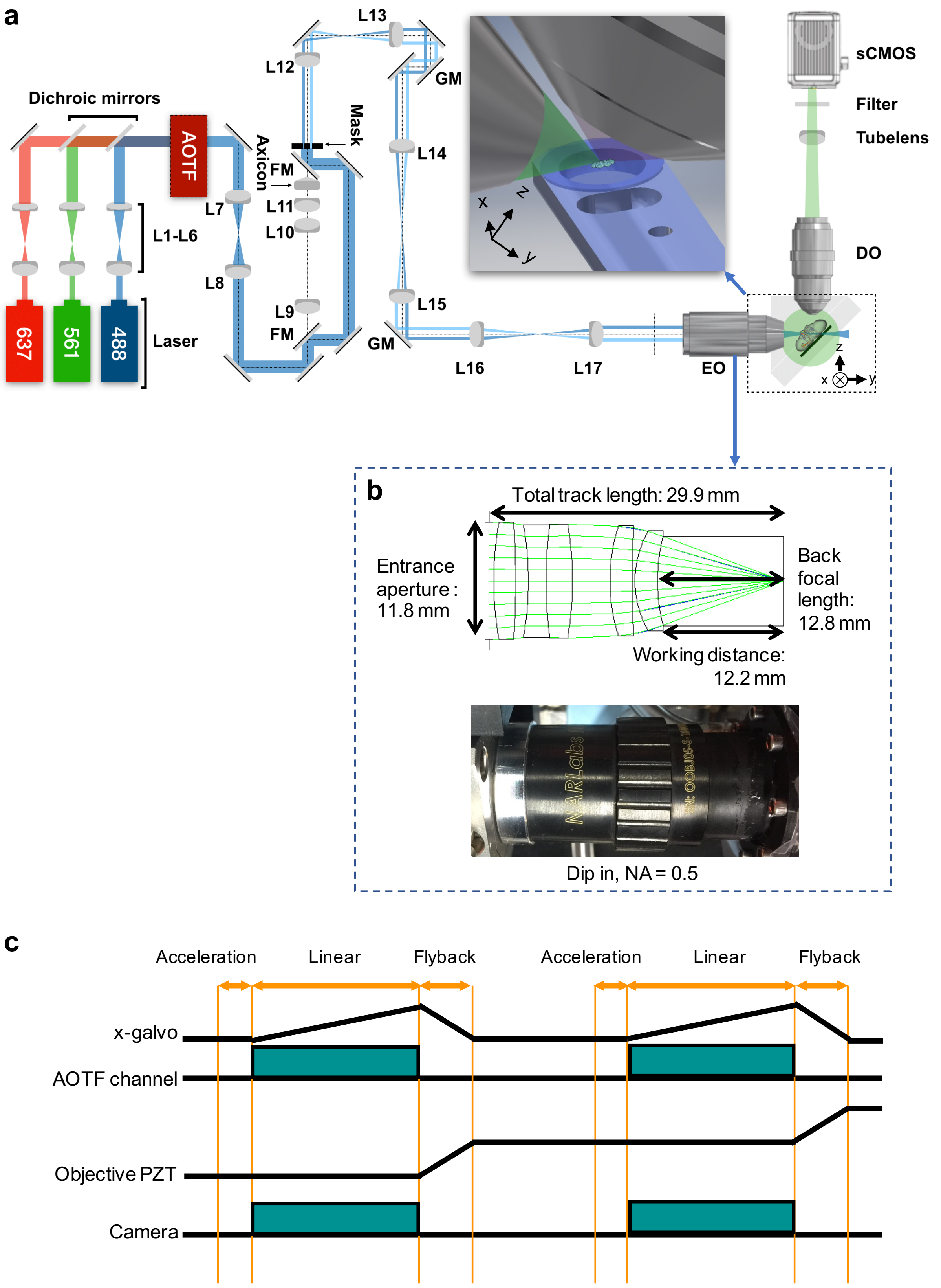

Supplementary Fig. 2. Additional beam path, excitation objective and waveforms in LLM-CT

(a) Schematic configuration of the mask-based LLM-CT system. AOTF, acoustic-optical tuneable filter; EO, excitation objective; DO, detection objective; GM, galvo mirror; FM, flip mirror, L, lens. (**b)** A customized oil dipping objective lens with a long working distance of 12.2 mm. Upper panel: Optical design and simulated ray tracing. Lower panel: Photograph of the lens. **(c)** Galvo and piezo waveforms. Acceleration, linear and flyback of the galvos and piezos (objective PZT) were controlled independently. Data acquisition occured during the linear ramp (constant velocity) portion of the waveforms.

Supplementary Fig. 3

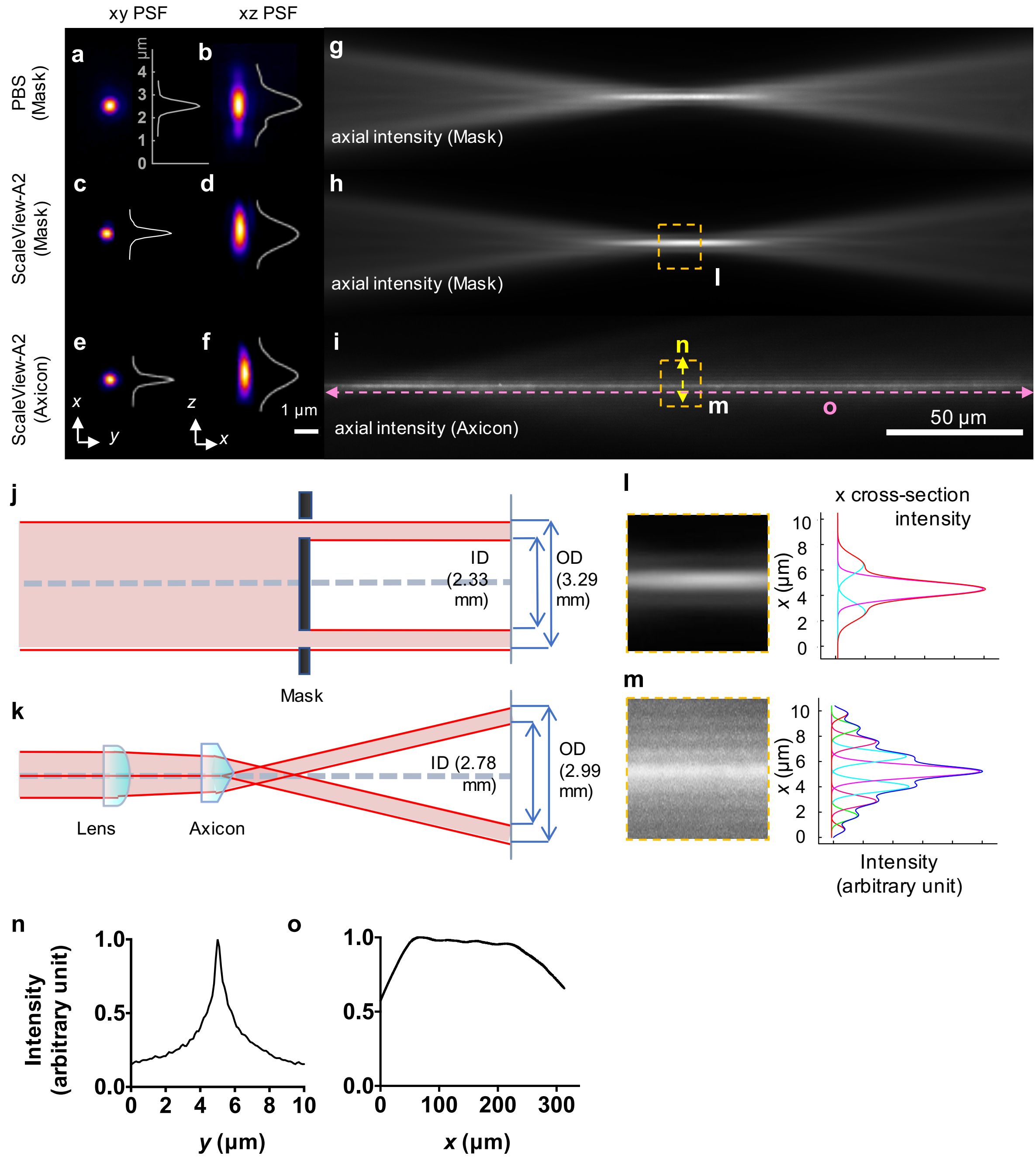

Supplementary Fig. 3. PSF and excitation Bessel beam profiles

(a-f) The point-spread function (PSF) acquired by Bessel lightsheet using the annular ring mask in phosphate buffered saline (PBS) (**a, b**)**,** in ScaleView-A2 (**c, d)** and by the axicon lens in ScaleView-A2 (**e, f)** observed from the X-Y and X-Z planes, respectively. Scale bar = 1 μm. **(g-i)** The axial profile of Bessel beam created by the mask in PBS (**g**), in ScaleView-A2 (**h)** and by the axicon lens in ScaleView-A2 (**i**), respectively. Scale bar = 50 μm. **(j-k)** Schematic illustrations of the Bessel beam generated using the mask (**j**) and axicon lens (**k**). The figures are not drawn to scale. **(l, m)** Detail illumination profiles and fitted intensity distributions of Bessel beams generated using the mask **(l)** and axicon lens **(m)** enlarged from the highlighted boxes in **h** and **i**, respectively. (**n-o**) Normalized intensity distributions along the x **(n)** and y **(o)** directions, respectively, of axicon lightsheet (line cuts indicated in i).

Supplementary Fig. 4

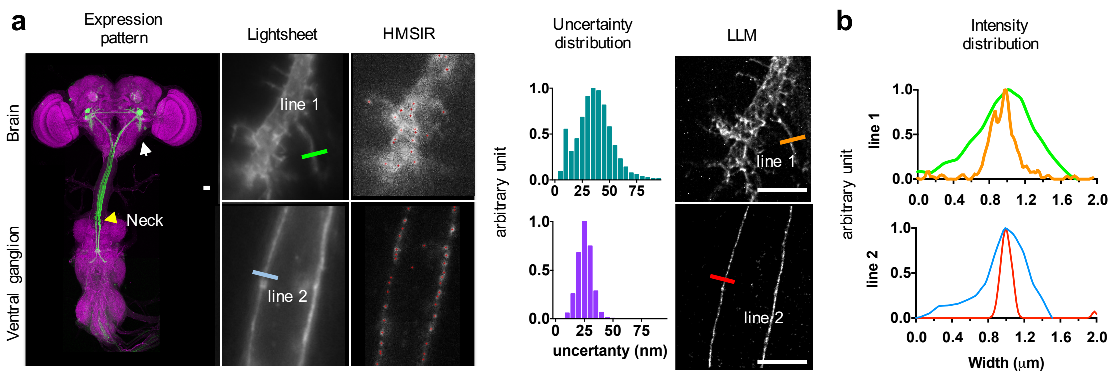

Supplementary Fig. 4. HMSiR localization precision between LM and LLM in different depths

(**a**) Paired giant-fiber neurons (green) in central nervous system (magenta) of a transgenic *12862-Gal4>UAS-mCD8::GFP* fly. A large-scale fluorescence image is shown to denote the relative position of the imaged subareas. The detailed images taken by lightsheet microscopy quantify GFP and HMSiR levels in dendrites in the whole brain (white arrow at the right, z = 53 µm) and a giant axon (yellow arrow at the left, z = 25 µm). HMSiR molecules (as shown as red dots) are labelled via anti-GFP immunolabeling. Uncertainty distribution: lateral uncertainty of localization events from the brain (top) and neck (bottom). LLM images of the same optical section examined using lightsheet microscopy. (**b**) Intensity distributions along the section denoted indicate that the width of the structural feature observed is significantly narrower for LLM than for lightsheet microscopy (two-fold and four-fold in the brain and neck, respectively). Colored lines indicate the measured locations. Scale bars = 10 µm.

Supplementary Fig. 5

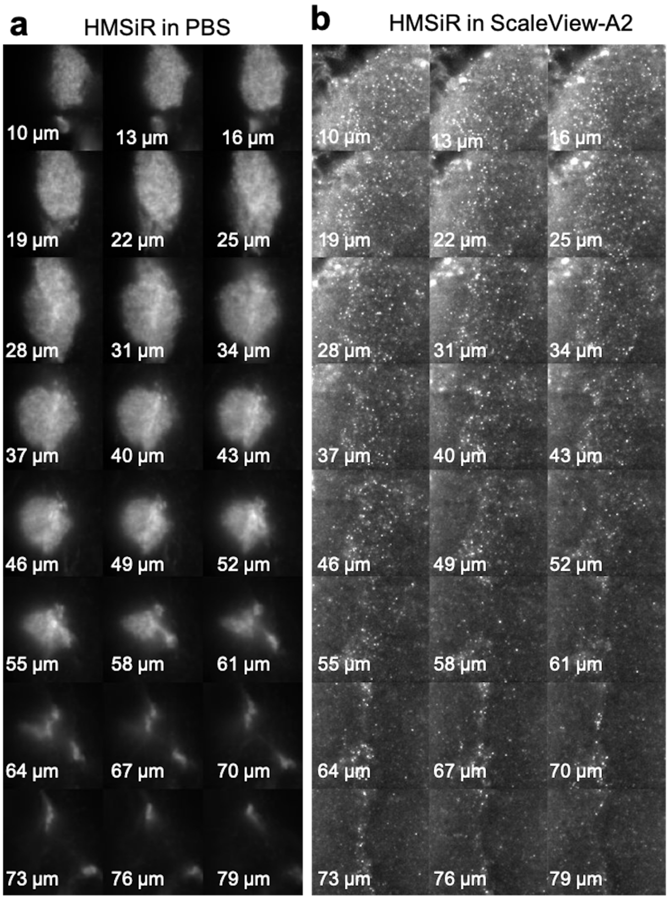

Supplementary Fig. 5. HMSiR blinking at different optical depths in PBS or Scaleview-A2

(a) HMSiR blinking signal in phosphate buffered saline (PBS) or **(b)** in ScaleView-A2 across different depths. HMSiR labeled membrane GFP in olfactory projection neurons in *MZ19-Gal4>UAS-mCD8::GFP* transgenic flies. Images were taken either under a 25× lens (Nikon, CFI Apo LWD 25XW, 1.1 NA, 2 mm WD) for PBS or a 25× objective lens (Olympus, XLPLN25XSVMP2, N.A. = 1.05, 2 mm WD) specifically designed for use with ScaleView-A2.

Supplementary Fig. 6

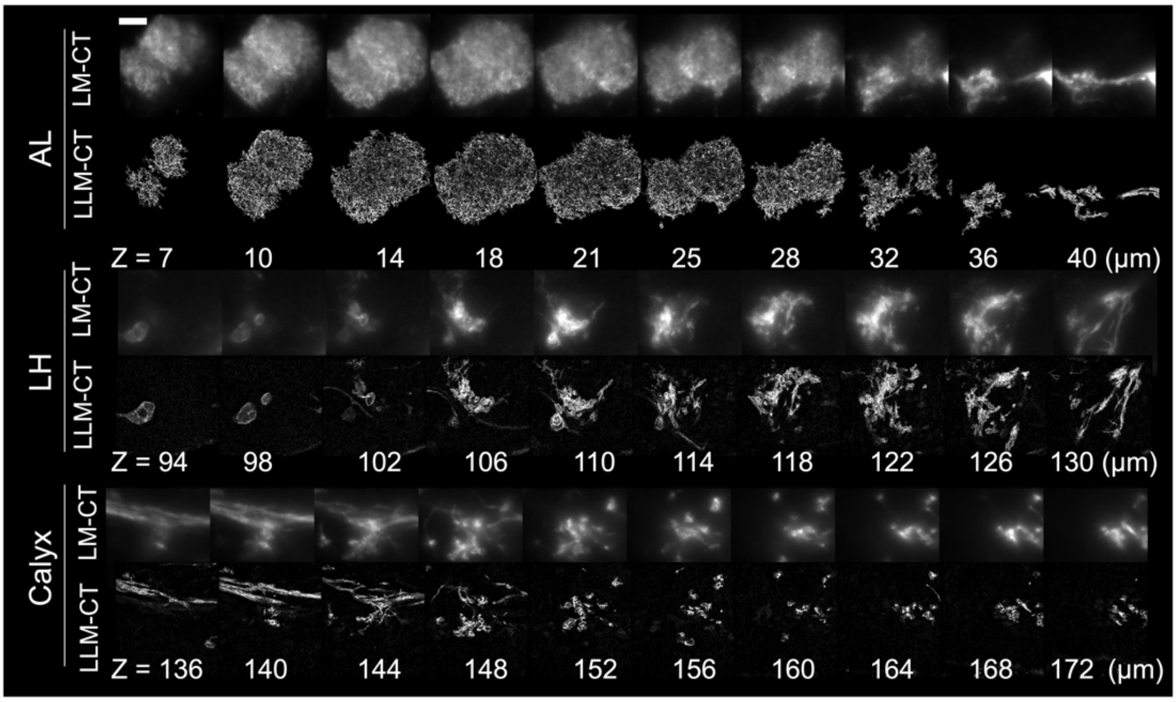

Supplementary Fig. 6. Comparing LLM-CT and LM-CT on MZ19-Gal4 labeled neurons

Single slide comparison of projection neuron images at different depths. Antennal lobe (AL, imaging depth: 0-66 µm), lateral horn (LH, imaging depth: 90-144 µm) and calyx (imaging depth: 129-189 µm) taken via Bessel lightsheet microscopy or Bessel lightsheet localization microscopy for clarified tissue (LM-CT or LLM-CT). Scale bar = 20 µm.

Supplementary Fig. 7

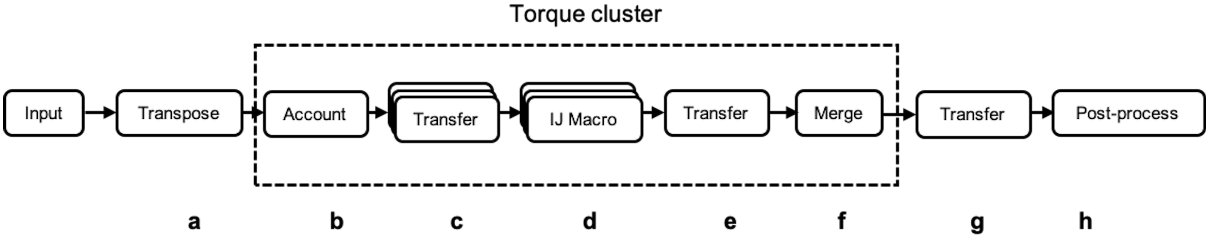

Supplementary Fig. 7. Flow chart of data processing for localization images.

(**a**) To perform drift correction in a single attempt, ThunderSTORM requires the user to provide a three-dimensional time series TIFF stack. Hence, XYZ stacks were converted to XYT stacks post-experiment on the acquisition workstation. A straightforward C program was used to parse the file via libtiff and re-write the data. (**b**) Accounting was performed on the head node of the cluster. After the file list was acknowledged, the data were evenly distributed to multiple parts, which was determined by the number of available worker nodes. Partial file lists were assigned to each worker node for data transfer. (**c**) The partial file list was presented to the rsync utility and copied from the local NAS to a temporary shared workspace (between worker nodes) created under the home directory of the worker node, because /tmp space was not large enough to store each batch of data. Additional wrapper script, an IJ macro and a calibration file (required for 3D localization) also were copied from the local NAS to worker nodes, to provide a minimalistic method for updating scripts on the cluster. The additional transfer time introduced was negligible. (**d**) As IJ remains "buggy" in headless mode, an XVFB (X-virtual framebuffer) and an in-memory display server are created by each worker node to contain each IJ instance. IJ read the user preference file during initialization, creating the file if it could not locate one. As this caused a race condition and put IJ in an unpredictable state, the user preference file was redirected to each workspace using JVM command line arguments. A temporary directory for the CSV results then was created under /tmp to avoid excessive small batch data transfer. This directory was removed upon termination of the JVM instance by a shell script trap. The macro detected the existence of a calibration file, called the respective 2D/3D configuration for ThunderSTORM and executed the appropriate function for all files in the workspace. CSV files were dumped on a per file basis; hence, each Z slice was associated with a respective output. (**e**) CSV files then were re-synchronized back to the shared workspace for merging. (**f**) The first worker, determined randomly by the head node during resource request, executed a Python script to merge the CSV files. An SQLite database was created in real time to hold all CSV files of interest. For 2D localization, sequential Z info was deduced based on Z step size and source file, because each Z slice was contained in a single 3D stack (see "Transpose"). NaN filtering and geometry manipulation, required by data which were acquired through sample scanning, also were performed at this stage. A new CSV file then was generated by the final content in the database. (**g**) The resulting CSV was re-synchronized back to local NAS storage under the source directory for further processing. (**h**) The resulting CSV then was loaded into ThunderSTORM to plot 2D/3D super-resolved images.

Supplementary Fig. 8

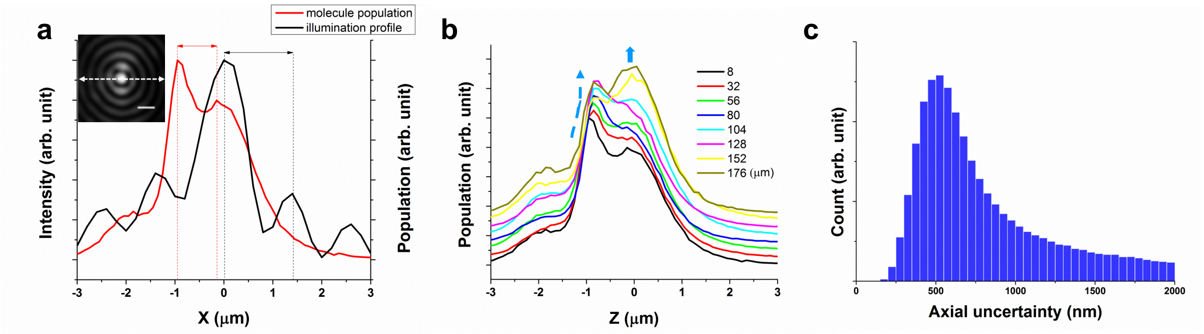

Supplementary Fig. 8. Error observed in the astigmatism defocusing model

(a) The excitation profile of the axicon-generated Bessel beam was (black line) compared with the axial population of molecules localized in LLM-CT (red line). Scale bar = 1μm. **(b)** The axial molecule distribution observed at different imaging depths. **(c)**. The distribution of axial localization uncertainty at the imaging depth = 8 μm.

Supplementary Fig. 9

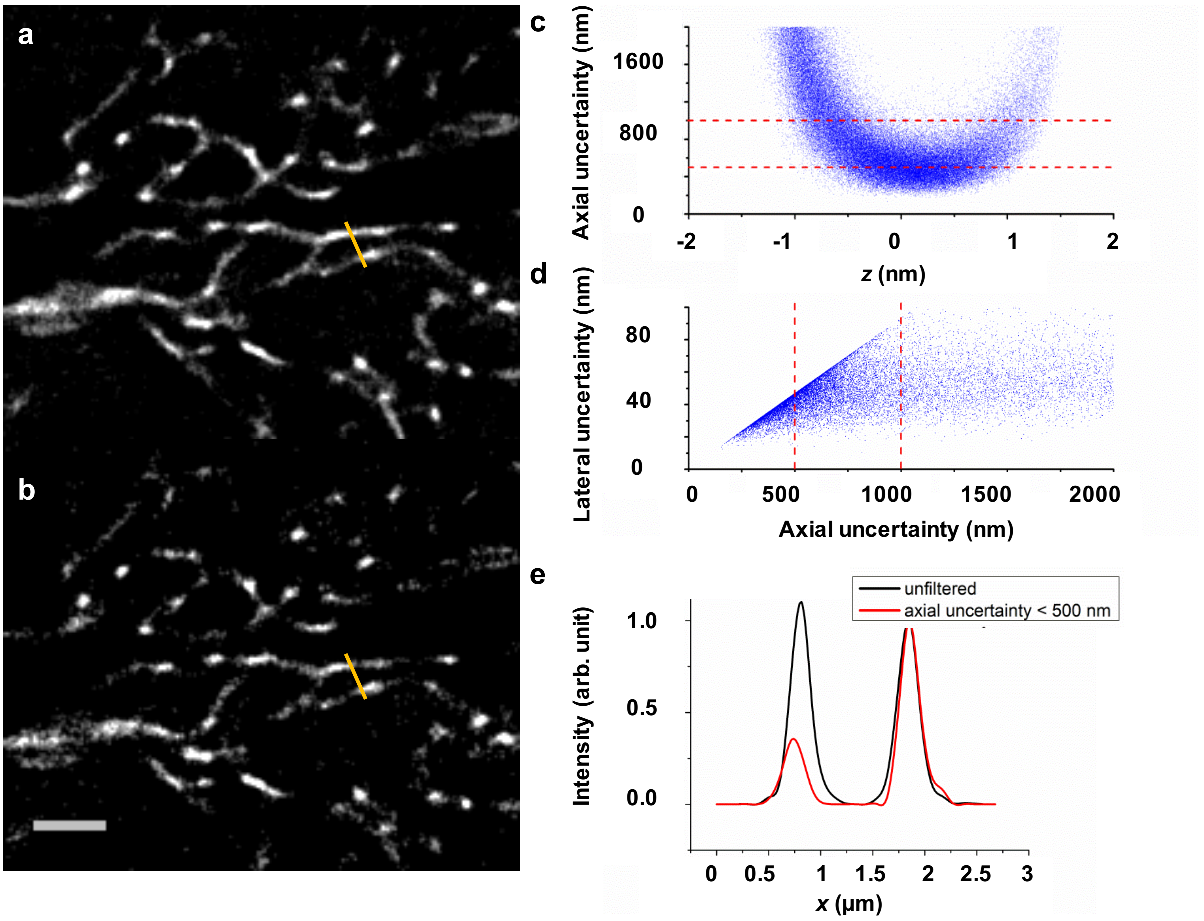

Supplementary Fig. 9. Improve image quality with axial localization uncertainty as a filter

(a) The original image of neurite fibers and **(b)** a filtered image of the same neurite fiber with a threshold of 500 nm axial localization uncertainty. **(c)** Axial uncertainty distribution was plotted versus axial position derived from the defocusing model. Red dotted lines denote a axial localization uncertainty threshold of 1000 nm (above) and 500 nm (below), respectively. **(d)** Correlation between lateral and axial localization uncertainty. Two red dotted lines denote thresholds of 1000 nm (right) and 500 nm (left), respectively. **(e)** Comparison of line profiles along the yellow segment shown in (a) and (b).

Supplementary Fig. 10

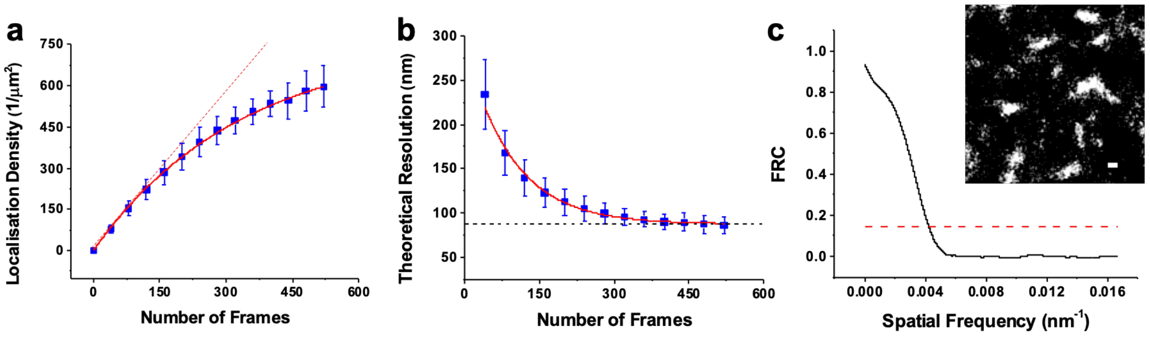

Supplementary Fig. 10. Analysis of localization density and resolution

(a) Localization density (number of events per square micrometer) increases exponentially with respect to the number of frames used in reconstruction. The red dotted line in a is a guide of eye which indicates the linear increase of the localization density (when there is no photobleaching presented). **(b)** The theoretical resolution increases rapidly as it reaches 100 nm. Resolution does not show a significant increase thereafter and finally ceases at 86 nm. The dotted line denotes the lower bound of the theoretical resolution achieved within 500 frames. **(c)** Fourier ring correlation (FRC) analysis of VMAT protein localization. The FRC value at the 1/7 cut-off frequency is 283.6±69.9 nm. The dotted line is the indicator of 1/7 cut off of FRC value. Inset in (c): a subarea-reconstructed super-resolution image of VMAT protein localization. Protein distribution presented an "island" structural feature. Each value represents mean ± S.D. (n = 4 in a, b; n = 3 in c). Scale bar = 100 nm.

Supplementary Fig. 11

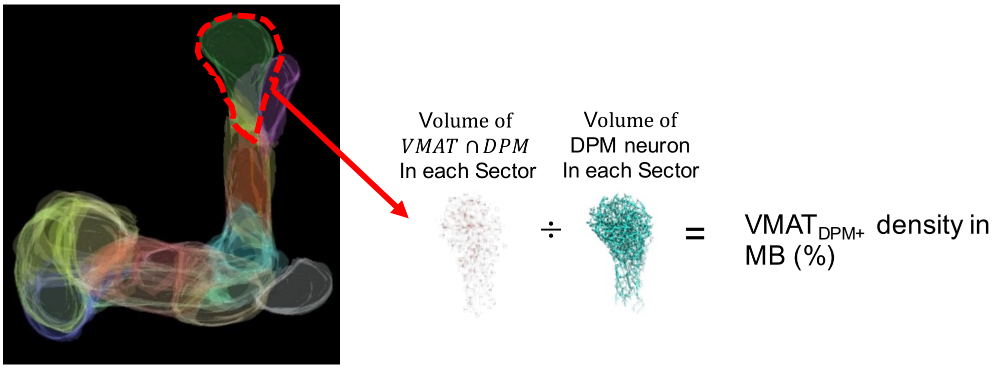

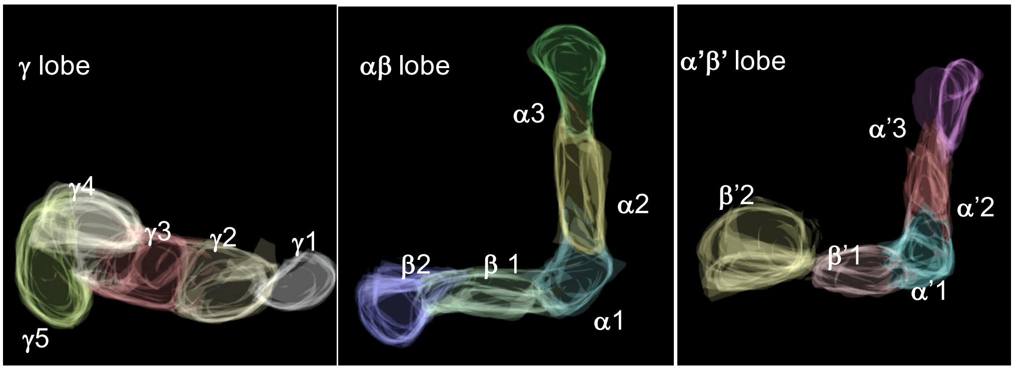

Supplementary Fig. 11. Measurement of VMAT density in MB lobe sectors

The MB lobes are subdivided into 15 sectors based on the input innervations from dopaminergic neurons (DAN). Right: schematic representations of the calculation process of VMAT density within (+) the DPM neuron in each MB sector. Bottom panel: Nomenclature of 15 MB sectors along the axonal bundles of the three MB lobes: γ (γ1– γ5),), α/β (α1, α2, α3, β1, β2) and α′/β′ (α′1, α′2, α′3, β′1, β′2).

Supplementary Fig. 12

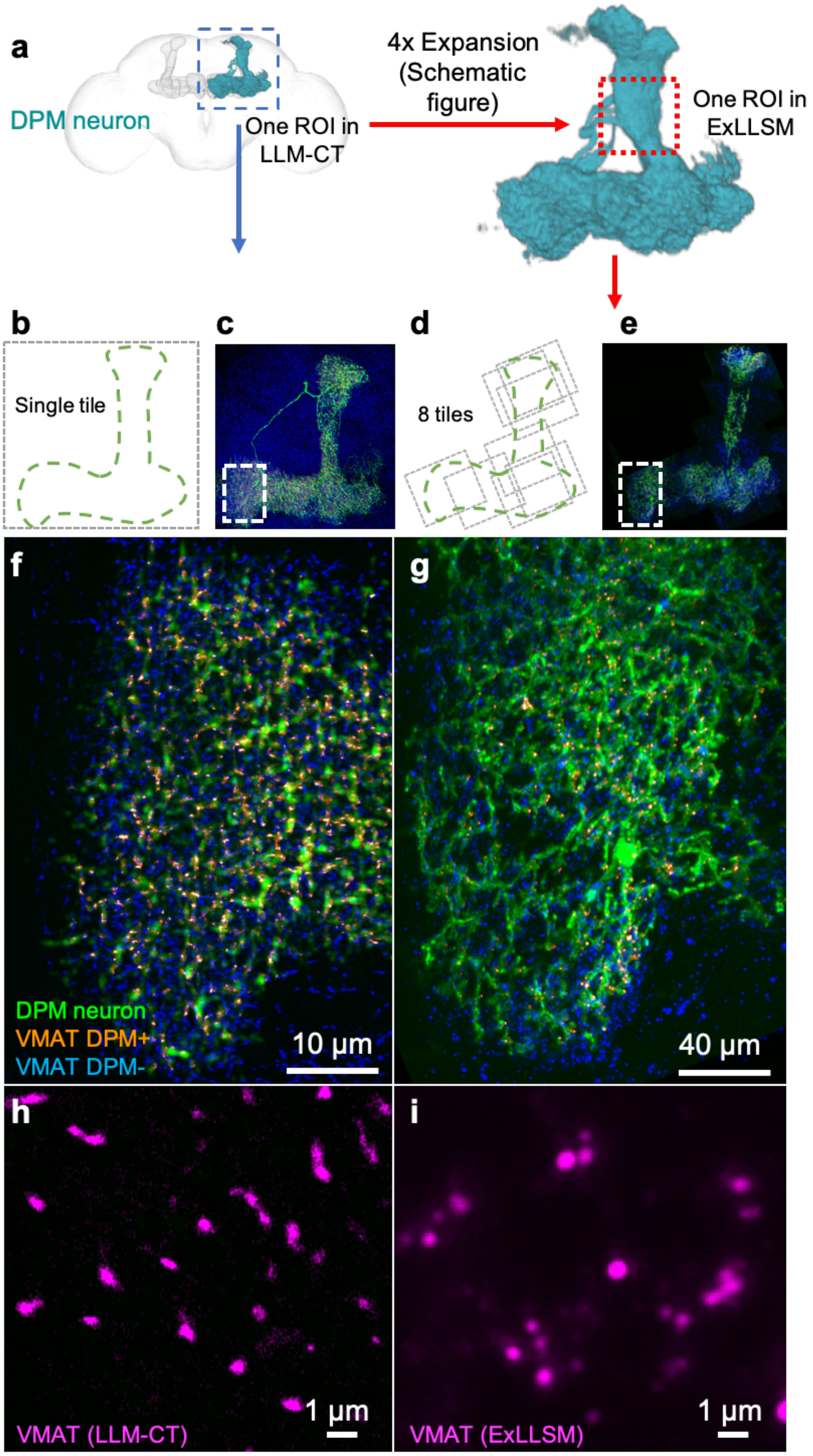

Supplementary Fig. 12. Comparison between images taken by LLM-CT and ExLLSM. (**a**) A DPM neuron occupied around 150 x 140 x 100 μm^3 volume in the fly brain (left). Expansion microscopy enlarged the entire DPM neuron 4X in x, y and z directions, 64 times in volume (right). (**b, c**) LLM-CT image of all VMAT molecules in the entire DPM neuron taken by a single optical volume. (**d, e**) ExLLSM image of the DPM neuron from stitching 8 tiles of volume image. (**f, g**) Enlarged volume images of the insets in c and e, respectively. Single-molecule localization of LLM-CT image (**f**) revealed numerous VMAT signals (orange) in DPM neurites (green) as well as VMAT signals outside DPM neurites (blue). Image resolution is comparable between ExLLSM (**g**) and LLM-CT (**f**), allowing reliable allocation of VMAT signals within or outside DPM neurites. (**h, i**) Enlarged images show significantly more DPM+ VMAT molecules captured with LLM-CT than ExLLSM. VMAT was immunostained with HMSiR (LLM-CT) or Alexa 635 (ExLLSM). Scale bars were indicated in the figure.

Supplementary Fig. 13

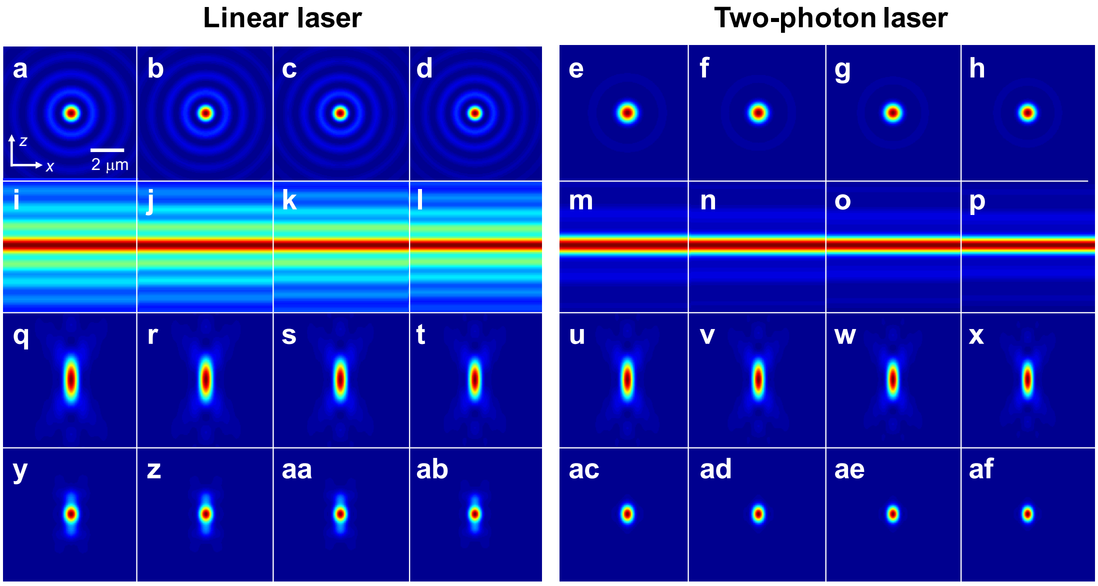

Supplementary Fig. 13. The corresponding PSFs by linear/two-photon laser at different n.

(**a-h**) Cross-sectional linear or two-photon excitation profiles of Bessel beams on focal plane at $n = 1.33$, 1.38, 1.45 and 1.52, respectively. The excitation wavelength is set to 0.637 μm (linear) or 1.04 μm (two-photon), and inner and outer impinging angles of the Bessel beam are fixed to those corresponding to numerical apertures of 0.5 and 0.42, respectively, at the back aperture of excitation objective lens. Because two-photon excitation was proportional to the square of intensity, the concentric rings on the excitation pattern of Bessel beams are dim. (**i-p**) Lightsheet (excitation PSFs) corresponding to the excitation profiles in a-h along the x direction. The main peak and side lobes on the sheets were related to the central peak and concentric rings in a to h. Side lobes from m-p are much less prominent than those in i-l due to the square dependence of 2-photon excitation. (**q-x**) Detection PSFs corresponding to a single HMSiR fluorophore in ambiances with various indices of n. The fluorescence wavelength was set to 0.66 μm (linear) or 0.58 μm (two-photon). The detection PSF is modeled as the field distribution of an objective lens whose NA is 1.1 when immersed in water ($n = 1.33$) near its focal point. The collection angles corresponding to these detection PSFs were set identical. (**y-af**) The overall PSFs in ambiances of different n. The overall PSFs were products of the excitation PSF (i-p) and the detection counterparts (q-x). The long tails in the axial ($\pm z$) directions are traces of side lobes on Bessel lightsheet. As the ambience index n became larger, the effective wavelength λ/n was reduced. This reduction effectively reduced the characteristic size of Bessel beams and detection PSFs. As a result, the overall PSFs became more compact in an environment with the higher refractive index. In the case of two-photon detection, no significant tails extended in the axial direction (ac-af in contrast to y-ab). All patterns related to two-photon detection scheme also were more localized and sharper in ambiances with higher refractive indices.

Supplementary Fig. 14

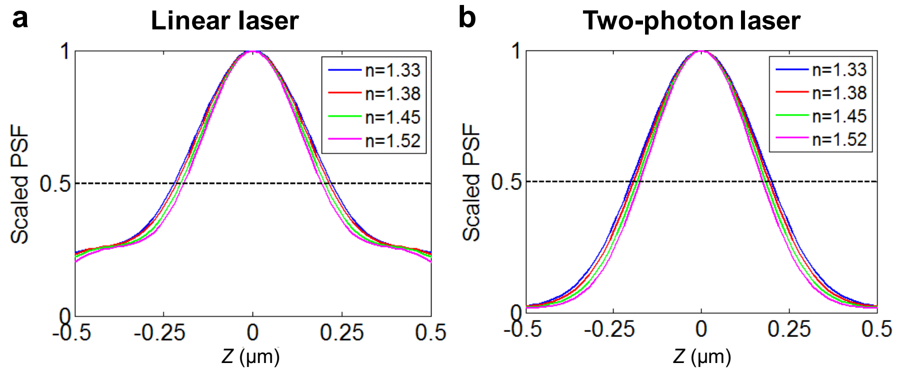

Supplementary Fig. 14. Scaled axial (*z*) distributions of overall PSFs at different *n*

The full width at half maximum of the overall PSF decreases with refractive index (n) under **(a)** linear excitation or **(b)** two-photon excitation, indicating a better axial resolution for single-molecule detection. The shoulders at two sides of the main peak are related to the background intensity of Bessel lightsheet.

Supplementary Fig. 15

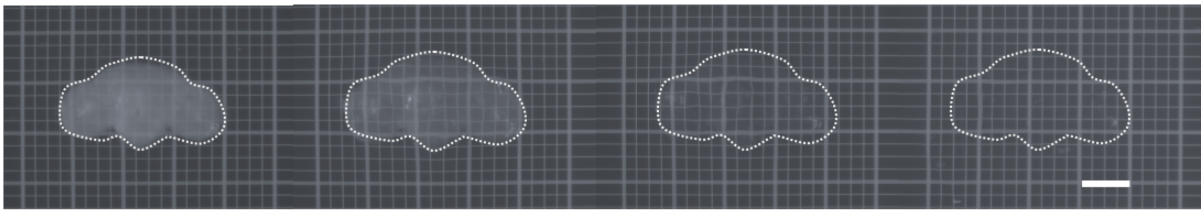

Supplementary Fig. 15. Effects of optical tissue clearing with different refractive index (RI) clearing reagents.

The same *D. melanogaster* brain was imbedded in different clearing reagents for 10 min before taking picture. Higher RI medium can make the brain most transparent. Scale bars = 200 μm.

Supplementary Table 1. Acquisition and image processing parameters for images in figures

Image property	Fig. 2b,	Fig. 2c	Fig. 2d	Supplementary Fig. 4	Supplementary Fig. 5a	Fig. 3, Supplementary Fig. 5b, 6 (AL)	Supplementary Fig. 6 (LH)	Supplementary Fig. 6 (Calyx)	Fig. 4a	Fig. 4b	Fig. 5, Supplementary Fig. 9	Fig. 6b, d-h
Sample	MZ19-Gal4>UAS-mCD8::GFP	MZ19-Gal4>UAS-mCD8::GFP	MZ19-Gal4>UAS-mCD8::GFP	12862-Gal4>UAS-mCD8::GFP	MZ19-Gal4>UAS-mCD8::GFP	MZ19-Gal4>UAS-mCD8::GFP	MZ19-Gal4>UAS-mCD8::GFP	MZ19-Gal4>UAS-mCD8::GFP	Fru-Gal4>UAS-mCD8::GFP	12862-Gal4>UAS-Dscam::GFP	TH-Gal4>UAS-GCmMP6m	VT64246-Gal4>UAS-mCD8::GFP
HMSiR target	Anti-GFP, projection neurons	Anti-GFP, projection neurons	Anti-GFP, projection neurons	Anti-GFP, Giant fiber neurons	Anti-GFP, projection neurons	Anti-GFP, projection neurons	Anti-GFP, projection neurons	Anti-GFP, projection neurons	Anti-GFP, descending neurons	Anti-GFP, Dscam on giant fiber neurons	Anti-GFP, Dopamine neuron	Anti-VMAT, endogenous VMAT protein
Medium	PBS	ScaleView-A2	ScaleView-A2	PBS	PBS	ScaleView-A2	ScaleView-A2	ScaleView-A2	ScaleView-A2	ScaleView-A2	ScaleView-A2	ScaleView-A2
Excitation wavelength (nm)	637	637	637	637	637	637	637	637	637	637	637	637
Lightsheet length (µm)	55	55	55	55	55	55	55	55	55	55	337	337
Excitation N. A. (outer; inner) of the excitation objective	0.26; 0.19	0.26; 0.19	0.26; 0.19	0.26; 0.19	0.26; 0.19	0.26; 0.19	0.26; 0.19	0.26; 0.19	0.26; 0.19	0.26; 0.19	0.19; 0.17	0.19; 0.17
Astigmatism	No	No	No	No	No	No	No	No	No	No	Yes	Yes
Voxel volume (x, y, z nm)	31 x 31 x 300	31 x 31 x 300	31 x 31 x 300	31 x 31 x 300	31 x 31 x 300	31 x 31 x 300	31 x 31 x 300	31 x 31 x 300	31 x 31 x 300	31 x 31 x 300	42 x 42 x 400	31 x 31 x 400
Image volume (x, y, z µm)	52 x 52 x 0.3	52 x 52 x 60	52 x 52 x 60	35 x 35 x 30	52 x 52 x 60	52 x 52 x 60	52 x 52 x 54	52 x 52 x 54	52 x 52 x 19	52 x 52 x 40	700 x 250 x 208	160 x 140 x 251
Number of slides	1	1	180	50,500	241,200	241,200	126,700	90,500	76,500	90,900	1,042,000	175,700
Number of subunit	1 x 1 x 1	1 x 1 x 1	1 x 1 x 1	1 x 1 x 1	1 x 1 x 1	1 x 1 x 1	1 x 1 x 1	1 x 1 x 1	1 x 1 x 1	1 x 1 x 1	4 x 1 x 1	1 x 1 x 1
Subunit size (x, y, z µm)	52 x 52 x 0.3	52 x 52 x 0.3	52 x 52 x 60	35 x 35 x 30	52 x 52 x 60	52 x 52 x 60	52 x 52 x 54	52 x 52 x 54	52 x 52 x 19	52 x 52 x 40	175 x 250 x 208	160 x 140 x 251
Pixel number per frame (x, y)	512 x 512	512 x 512	512 x 512	352 x 352	512 x 512	512 x 512	512 x 512	512 x 512	512 x 512	512 x 512	1400 x 2000	1600 x 1400
Exposure time/frame (ms)	100	100	100	100	100	100	100	100	100	100	80	80
Volume speed (μm^3/sec)	N.A.	N.A.	N.A.	3,675	8,112	8,112	8,112	8,112	8,112	8,112	700,000	89,600
Imaging period (hr)	100 ms	100 ms	100 ms	1.4	6.7	6.7	3.5	2.5	1.7	2.5	23.2	4.8
Localized molecules	N.A.	N.A.	100,914	1,415,065	N.A	26,714,852	6,407,961	4,015,525	36,729,117	722,594	523,273,389	(Average)
Corresponding movie	N.A.	N.A.	N.A.	N.A.	N.A.	Supplementary Movie 1, 2	Supplementary Movie 2, 3	Supplementary Movie 2	N.A.	N.A	Supplementary Movie 4	Supplementary Movie 5

Supplementary Table 2. Comparison between LLM-CT and ExLLSM for super-resolution imaging of an adult fly brain.

	**LLM-CT**<br>**Lightsheet + Localization**	**ExLLSM**<br>**Lightsheet + Expansion**
Sample stating	Regular staining process with SA-HMSiR	Expansion protocol
Sample size for 1 adult fly brain	0.7 x 0.4 x 0.25 mm	~2.7 x 1.4 x 0.37 mm^3
Expansion factor	1	3.99
Mounting medium	Scaleview-A2	Water
Lighsheet type	Bessel lightsheet microscopy	Automated Lattice lighsheet microscopy
Imaging period for 1 adult fly brain per channel	~24 hrs	~ 31 hrs
Original imaged ROIs for single channel	4	25788
Original imaged tiles	2800 (700 repeated volume in one tile)	25788
Resolution	30*30*500 nm (average uncertainty for each blinking signal across z direction)	60*60*90 nm (when expansion factor ~ 4)
Image processing	Customized algorithms for import data into ThunderSTORM on the cluster (ImageJ).	Customized algorithms for flat-field correction, deconvolution and stitching.
Output image size	122 GB (voxel volume: 33 x 33 x 500 nm)	~700 GB (voxel volume: 23.7 x 23.7 x 44 nm)

Supplementary Table 3. Volumes, total imaging times, predicted numbers of molecules and estimated processing times needed to apply our LLM-CT method to multi-scale samples.

Sample type	Dendrites of 10 olfactory neurons in 3 glomeruli in antenna lobe of intact fly brain	Dopaminergic neurons in the whole fly brain	Mouse brain	Human brain
Volume (mm^3)	1.6×10^{-4}	5.6×10^{-2}	5.1×10^{2}	1.3×10^{6}
Total imaging time	6.7 hours	23.2 hours	2.6 years (estimated)	6.4 x 103 years (estimated)
Localized molecules	2.6×10^{7}	5.2×10^{8}	7.7×101^{2} (estimated)	1.9×10^{16} (estimated)
Processing time	1.2 hours	24 hours	40.5 years (estimated)	105 years (estimated)

Imaging times and number of molecules were scaled for mouse brain[2] and human brain[3] based on the volume of an adult fly brain. The estimations of processing time were calculated by the average processing time for localizing one single molecule in our cluster (200 µsec) and the number of localized molecules in the specified sample.

Supplementary Note 1. Using an astigmatism defocusing model to improve optical sectioning and lateral resolution.

The cross section of Bessel beam in the inset of **Supplementary Fig. 8** showed a concentric intensity distribution. Along the segment in the lateral direction of the cross section, the 1D intensity distribution was represented as a black line. Most of the laser energy was confined in the central lobe, whereas the rest of the energy was periodically distributed into the side lobes because of interference between the wave vectors. The beam profile was measured by scanning the laser two dimensionally across a 200-nm fluorescence bead (Thermo Fisher, F8807) in ScaleView-A2 with no sample present.

The axial position distribution of the molecules localized within the *TH-Gal4* sample at the imaging depth of 8 μm was plotted (red line) with the 1D intensity distribution of the Bessel beam. The axial position of the molecules was fitted by using the astigmatism defocusing model implemented in ThunderStorm[4, 5]. As indicated by the red line shown in **Supplementary Fig. 8a**, the axial distribution of localized molecules exhibited three primary peaks, which were excited by the central lobe and the first side lobes, respectively, from the illumination profiles. The populations of molecule distributions were asymmetric, which differed from the relatively symmetric excitation profile. An offset of the primary peak from the illumination profile and a decrease in the distance between the central and the second lobes was observed. The difference between the two distribution curves most likely was related to the remaining refractive index mismatch in the optical clarified tissue.

Further investigation was conducted on the aberrant molecule distribution profiles related to imaging depth. Shown in **Supplementary Fig. 8b**, a blue-shift of the position of the primary peak and an increase in the population of the peak at the right-hand side were observed as the imaging depth increased. This indicated an imaging depth-dependent aberration existed in the sample, which re-distributed the excitation profile within the sample. The calibration curves thus generated from fluorescence beads failed to fit the PSF within the sample without considering this sample induced aberration. This effect caused a systematic error when applying the defocusing model to fit the axial position of the molecules within the tissue and lead to a large axial uncertainty distribution (**Supplementary Fig. 8c**).

Although the relatively large axial localization uncertainty hindered the resolution of axial position with sub-diffraction resolution, axial localization uncertainty still could use during post processing as a threshold to filter out the molecules excited by the side lobes. As shown in **Supplementary Fig. 9c**, the axial localization uncertainty was distributed parabolically with respect to axial position. By setting a threshold, the molecules with an axial localization uncertainty higher than the specified value could be excluded from image reconstruction. In **Supplementary Fig. 9a**, the original image without setting the threshold is presented. After applying a threshold of 500 nm for axial localization uncertainty, a filtered image was shown in **Supplementary Fig. 9b**. This filtering procedure not only ensured that the axial position is within the central lobe of the Bessel beam, but also refined the lateral localization uncertainty of the localized molecules. The dependence between the lateral and axial localization uncertainties was plotted in **Supplementary Fig. 9**. A threshold of axial uncertainty at 500 nm also suppressed the upper bound of lateral uncertainty, which intrinsically improved lateral resolution. Line profiles of segments shown in **Supplementary Fig. 9a** and **b** were compared in **Supplementary Fig. 9e.** Line profiles of the filtered image

showed different heights for the first peak, which suggested that the localized molecules observed within the first peak in the unfiltered image resulted from out-of-focus signal (excited by the side lobes). After filtering, the peak width of the first peak became significantly narrower because molecules with poor lateral localization uncertainty were removed. Compared to the original image, moreover, the neurite fibers presented in **Supplementary Fig. 9b** showed less blurry parts from the out-of-focus signal and clearer fiber tracks. Such improvements were essential for segmentation of axially overlapped structures and increased accuracy for proper identification of neurite morphology.

Supplementary Note 2 - Analysis of image acquisition speed and resolution.

To evaluate the imaging performance of VMAT protein expression in DPM neurons, a 2D resolution analysis was performed. As the number of sampling frames increased, growth of localization density slowed because of photo-bleaching and the depletion of fluorophores (**Supplementary Fig. 10a)**. Theoretical resolution increased rapidly in the first 300 frames (**Supplementary Fig. 10b)** and converged at 86 nm, which represents the lower bound resolution limit. It is worth comparing the FRC analysis with the structural information obtained from the VMAT protein localization. The FRC value at a 1/7 cutoff frequency in VMAT protein localization was 283.6± 69.9 nm (**Supplementary Fig. 10c)**, which is significantly larger than the theoretical resolution. As shown in the inset, the VMAT protein tended to condense into puncta with an irregular shape and a size distribution around hundreds of nanometers. The size distribution of VMAT protein islands tended to explained the higher observed FRC value and the disagreement with theoretical resolution. Co-localization analysis of VMAT proteins with DPM neuron (**Fig. 6**) depended on a priori knowledge that VMAT proteins were in vesicles distributed within or in proximity to neurons. Consequently, the reliability of results was dominated by the localization precision of each single molecule rather than any structural features of the molecular ensembles. According to the analysis described in the last paragraph, we thus kept the number of frames of time-lapse data used in VMAT localization to 400-500 per layer, which was enough to resolve the VMAT distribution. Statistics were made based on similar localization density and theoretical resolution for comparison. Acquisition time of one VMAT dataset thus was about 5.6 hour (100 msec per frame, 401 frames per imaging volume, 500 volume recorded). For localization of anti-TH signals (**Fig. 5**), acquisition time was 7.2 hour (100 msec per frame, 521 frames per imaging volume, 500 volume recorded). With four subvolumes per whole brain, total acquisition time was 28.8 hours.

Supplementary References:

1. Gao R*, et al.* Cortical column and whole-brain imaging with molecular contrast and nanoscale resolution. *Science* **363**, eaau 8302 (2019).
2. Badea A, Ali-Sharief AA, Johnson GA. Morphometric analysis of the C57BL/6J mouse brain. *Neuroimage* **37**, 683-693 (2007).
3. Allen JS, Damasio H, Grabowski TJ. Normal neuroanatomical variation in the human brain: an MRI-volumetric study. *Am J Phys Anthropol* **118**, 341-358 (2002).
4. Huang B, Wang W, Bates M, Zhuang X. Three-dimensional super-resolution imaging by stochastic optical reconstruction microscopy. *Science* **319**, 810-813 (2008).
5. Ovesny M, Krizek P, Borkovec J, Svindrych Z, Hagen GM. ThunderSTORM: a comprehensive ImageJ plug-in for PALM and STORM data analysis and super-resolution imaging. *Bioinformatics* **30**, 2389-2390 (2014).
