# Supplementary material for: Rapid single-wavelength lightsheet localization microscopy for clarified tissue
Source: Nat Commun. 2019 Oct 18;10:4762. doi: 10.1038/s41467-019-12715-3 (PMC6800451; doi:10.1038/s41467-019-12715-3)
Supplement: Supplementary file 3 — Description of Additional Supplementary Files [file 41467_2019_12715_MOESM3_ESM.pdf]

**Description of Additional Supplementary Files**

**File Name: Supplementary Movie 1**

**Description: Comparing image quality between LM-CT and LLM-CT.** Olfactory projection neurons labeled in <i>MZ19-Gal4</i> were imaged by LM-CT (top panels) and LLM-CT (bottom panels), respectively, from anterior (antennal lobe) to middle (lateral horn) and posterior (calyx) of the brain. Imaging depth is indicated. Scale bar = 10 µm.

**File Name: Supplementary Movie 2**

**Description: Stochastic blinking raw images and reconstruction process.** Left: 3D ortho slice of super-resolved dendritic <i>MZ19-Gal4</i> labeled olfactory projection neuron in three antennal lobe glomeruli. Middle: Time series of blinking events of one layer (z = 24 µm) projection neuron neurites immunostained with HMSiR. Right: Reconstruction process of localization events.

**File Name: Supplementary Movie 3**

**Description: 3D rendering of neurons imaged with LLM-CT.** 3D rendering of neurons located in lateral horn area in <i>MZ19-Gal4</i> labeled projection neurons in <i>D. melanogaster</i> brain imaged with LLM-CT.

**File Name: Supplementary Movie 4**

**Description: Whole-brain super-resolution image.** LLM-CT super-resolved dopaminergic neurons labeled in the <i>TH-Gal4</i>. Serial slices show well-resolved neurites throughout the whole brain, even at the optical lobe where fine neurites emit weak fluorescence.

**File Name: Supplementary Movie 5**

**Description: Super-resolved protein distribution in <i>D.** melanogaster</i> brain. Spatial distribution of VMAT proteins within and outside the DPM neurites in the whole MB.
