# Supplementary material for: Rapid single-wavelength lightsheet localization microscopy for clarified tissue
Source: Nat Commun. 2019 Oct 18;10:4762. doi: 10.1038/s41467-019-12715-3 (PMC6800451; doi:10.1038/s41467-019-12715-3)
Supplement: Supplementary file 9 — Reporting Summary [file 41467_2019_12715_MOESM9_ESM.pdf]

nature research

Corresponding author(s): Ann-Shyn Chiang, Bi-Chan Cheng

Reporting Summary

Nature Research wishes to improve the reproducibility of the work that we publish. This form provides structure for consistency and transparency in reporting. For further information on Nature Research policies, see Authors & Referees and the Editorial Policy Checklist.

Statistical parameters

When statistical analyses are reported, confirm that the following items are present in the relevant location (e.g. figure legend, table legend, main text, or Methods section).

n/a	Confirmed	
☐	☒	The exact sample size (n) for each experimental group/condition, given as a discrete number and unit of measurement
☐	☒	An indication of whether measurements were taken from distinct samples or whether the same sample was measured repeatedly
☐	☒	The statistical test(s) used AND whether they are one- or two-sided<br>*Only common tests should be described solely by name; describe more complex techniques in the Methods section.*
☐	☒	A description of all covariates tested
☐	☒	A description of any assumptions or corrections, such as tests of normality and adjustment for multiple comparisons
☐	☒	A full description of the statistics including central tendency (e.g. means) or other basic estimates (e.g. regression coefficient) AND variation (e.g. standard deviation) or associated estimates of uncertainty (e.g. confidence intervals)
☐	☒	For null hypothesis testing, the test statistic (e.g. F, t, r) with confidence intervals, effect sizes, degrees of freedom and P value noted<br>*Give P values as exact values whenever suitable.*
☐	☒	For Bayesian analysis, information on the choice of priors and Markov chain Monte Carlo settings
☐	☒	For hierarchical and complex designs, identification of the appropriate level for tests and full reporting of outcomes
☐	☒	Estimates of effect sizes (e.g. Cohen's d, Pearson's r), indicating how they were calculated
☐	☒	Clearly defined error bars<br>*State explicitly what error bars represent (e.g. SD, SE, CI)*

Our web collection on statistics for biologists may be useful.

Software and code

Policy information about availability of computer code

Data collection	Modified LatticeScope software was used in this study for localisation image collection. Zen was used in Zeiss microscope for collecting confocal/airyscan images.
Data analysis	Image J plugin ThunderSTORM was used to analyze blinking event to reconstruct localisation super-resolution images. Avizo 9.4 (Thermo Fisher Scientific Inc) was used to rendering neurons in all figures and used to calculate the protein density in Fig. 6. Excel was used to do all the statistics.

For manuscripts utilizing custom algorithms or software that are central to the research but not yet described in published literature, software must be made available to editors/reviewers upon request. We strongly encourage code deposition in a community repository (e.g. GitHub). See the Nature Research guidelines for submitting code & software for further information.

Data

Policy information about availability of data

All manuscripts must include a data availability statement. This statement should provide the following information, where applicable:

- Accession codes, unique identifiers, or web links for publicly available datasets
- A list of figures that have associated raw data
- A description of any restrictions on data availability

The data that support the findings of this study are available from the corresponding author upon reasonable request.

Field-specific reporting

Please select the best fit for your research. If you are not sure, read the appropriate sections before making your selection.

☒ Life sciences ☐ Behavioural & social sciences ☐ Ecological, evolutionary & environmental sciences

For a reference copy of the document with all sections, see nature.com/authors/policies/ReportingSummary-flat.pdf

Life sciences study design

All studies must disclose on these points even when the disclosure is negative.

Sample size	All image sample size are described in figure legends. Two-tail Student t test was used to determine the significant differences between paired groups.
Data exclusions	No data been excluded in this study.
Replication	All images experiments were done in more then one day. Data collected in multiple days were all been used for statistic analysis.
Randomization	All imaged flies were randomly choose from a group of flies that 100 times more then the group been used in this study.
Blinding	VMAT super-resolution images in this study were also been processed by 3 different group of people and all been used in the final comparison.

Reporting for specific materials, systems and methods

Materials & experimental systems

n/a	Involved in the study	
☒	☐	Unique biological materials
☐	☒	Antibodies
☒	☐	Eukaryotic cell lines
☒	☐	Palaeontology
☐	☒	Animals and other organisms
☒	☐	Human research participants

Methods

n/a	Involved in the study	
☒	☐	ChIP-seq
☒	☐	Flow cytometry
☒	☐	MRI-based neuroimaging

Antibodies

Antibodies used	Immunostaining was sequentially performed in PBS containing 1% Triton X-100 and 0.25% NGS using the following primary antibodies: mouse anti-Discs large antibodies (antibody 4F3; 1:20 dilution; Developmental Studies Hybridoma Bank, Iowa City, IA), rabbit anti-GFP antibodies (1:250 dilution; Thermo Fisher Scientific Inc, A11122), rabbit anti-VMAT (1:250 dilution).
Validation	Mouse anti-Discs large antibodies information is provided in the following website:http://dshb.biology.uiowa.edu/4F3-anti-discs-large; Rabbit anti-GFP antibody information is provided in the following website: https://www.thermofisher.com/antibody/product/GFP-Tag-Antibody-Polyclonal/A-11122; Rabbit anti-VMAT was provided by David Karntz (Brooks. et. al., 2011, Neuron),

Animals and other organisms

Policy information about studies involving animals; ARRIVE guidelines recommended for reporting animal research

Laboratory animals	Both male and female fruit flies (Drosophila melanogaster) of 5-7 day old were used in this study.
Wild animals	The study did not involve wild animals.
Field-collected samples	The study did not involve samples collected from the field.
